# Minimally invasive surgery in paediatric nephroblastoma

**DOI:** 10.3332/ecancer.2024

**Published:** 2025-11-13

**Authors:** Steven W Warmann, Jörg Fuchs

**Affiliations:** 1 Department of Paediatric Surgery and Paediatric Urology, Charité University Hospital, Berlin, Germany; 2 Department of Paediatric Surgery and Paediatric Urology, University Children’s Hospital, Tübingen, Germany

**Keywords:** nephroblastoma, minimally invasive surgery, tumour nephrectomy

## Abstract

Surgery is a corner stone of treatment in children with nephroblastoma. Over recent years, the evolution of surgical guidelines has added to the improvement of treatment results in affected children. Minimally invasive surgery (MIS) has been described as part of this evolution. The present article summarises the current recommendations for MIS in nephroblastoma based on the relevant global treatment protocols.

## Introduction

Minimally invasive surgery (MIS) has gained increasing importance in all fields of paediatric surgery, including surgical oncology. More and more treatment protocols for paediatric solid tumours include guidelines on MIS [1–3]. MIS has been used in paediatric urology relatively early, consecutively, renal tumours came into the focus of surgeons in this regard. As a consequence, historical reports on MIS for paediatric renal tumours occurred fairly early [4, 5]. The surgical panel of the SIOP Renal Tumour Study Group (SIOP RTSG) has for the first time systematically evaluated the results of MIS in paediatric nephroblastoma and has formulated guidelines, which are part of the current SIOP RTSG Umbrella protocol [6]. Meanwhile, there is a growing experience with this subject [7–9]. Surgical results and oncological outcomes of MIS are similar to the results obtained by open surgery; however, lymph node sampling is often insufficient when MIS is used [8, 10].

The use of robotic surgery has recently been introduced in children with nephroblastoma. Preliminary results indicate its potentially favorable contribution; however, the definitive role of this technique and its possible addition to treatment protocols still have to be clarified. Specific respective surgical guidelines seem necessary [11–13].

Taken together, MIS has been established as a safe and successful technique for resection of nephroblastoma provided there is careful patient selection, and surgeons have sufficient expertise in the fields of MIS, oncologic surgery and paediatric urology. Irrespective of the specific technique that is applied, the oncological principles of Wilms Tumour surgery have to be strictly respected.

## Indications and contraindications

Based on the experiences of the SIOP 2001 study, the SIOP RTSG formulated the following indications and contraindications for MIS in nephroblastoma in the current Umbrella Protocol [6, 14].

### Indications:

1. Resection must adhere to oncological principles and include lymph node sampling.
2. Small, central tumours with a rim of ‘normal’ renal tissue.
3. The extraction of the specimen in a bag, without morcellation, through an adequate abdominal wall incision, is mandatory, not only to control the risk of dissemination, but also to ensure adequate histopathological staging.
4. If nephron-sparing surgery (NSS) is feasible, it should be preferred even if an open approach is needed.

### Contraindications:

1. Tumour infiltrating extra-renal structures or extending beyond the ipsilateral border of the spinal column.
2. Thrombus in the renal vein or vena cava.
3. Peripheral location if NSS is not deemed feasible.
4. Tumour without any response to chemotherapy due to the risk of tumour rupture.
5. Little or no experience in laparoscopic nephrectomy (consider transfer to another unit or obtain more experienced help).

According to the SIOP Umbrella protocol, neoadjuvant chemotherapy is a precondition for MIS. Provided there is a careful patient selection, MIS for nephroblastoma is associated with comparable results as the open approach regarding complications, surgical results, and oncological outcome. Current analyses imply that not every child with Wilms Tumour is per se a candidate for MIS. This holds even more true if upfront surgery is recommended as the primary approach, as is, for example, the case for certain conditions in the protocols of the US-based Children’s Oncology Group.

The SIOP RTSG guidelines for MIS in children were introduced in a rather conservative manner, with the main focus on their applicability and safety. Since their introduction, some authors have reported that experienced surgeons may address some of these aspects with a broader view concerning indications [15].

Minimally invasive NSS for nephroblastoma has not been systematically analysed so far. There exist some reports on singular cases. Authors in this regard regularly formulate the necessity for caution and the need to further analyse this approach before its advocation on a larger scale [16, 17].

## Surgical approach – minimally invasive tumour nephrectomy

Currently, the surgical approach of MIS in nephroblastoma can be divided into three groups: transperitoneal laparoscopic, retroperitoneal or robotic. To date, there exists no systematical analyses comparing these three MIS approaches. The largest number of authors reported on the usage of the transperitoneal approach. According to the Umbrella protocol of the SIOP RTSG, children with nephroblastoma have to receive neoadjuvant chemotherapy before undergoing MIS. Concerning patients' age, MIS can be executed safely after chemotherapy, irrespective of the age and size of the children.

For the laparoscopic approach, usually a three- or four-trocar technique is used with the children being positioned in a supine or an angled decubitus position [8, 9]. Mobilisation of the colon on the affected side is followed by mobilising of the organ and exploration of the vessels. This step can relevantly be facilitated by using a transabdominal traction suture through the proximal ureter to elevate the medial plane of the kidney and the renal hilus. The rule of transsecting the renal artery before the vein applies to MIS identically. Lymph node sampling should be performed in accordance with the guidelines of open surgery. In some cases, it can be preferable to perform lymph node sampling before dividing the renal vessels. The reason for this is the potential retraction of midline structures after vascular transection, through which identification of lymph nodes and regions of their origin might become more difficult. The ureter should be divided as closely as possible to the urinary bladder. After complete mobilisation, the resected specimen should be removed from the situs without morcellation, preferably using a retrieval bag. Most authors advocate a Pfannenstiel incision for the removal of the specimen.

Slight alterations of the approach have been reported when using the retroperitoneal approach. These alterations mainly concern a larger angle for patient positioning, positioning of trocars, establishment of a retroperitoneal space, and exploration of the targeted organs [18, 19].

In conclusion, MIS for paediatric nephroblastoma is an established technique with a distinct role in the surgical treatment of affected patients. Patient selection and surgical expertise are the most important factors to grant results that are comparable with those of open surgical procedures. The role of minimally invasive NSS and robotic surgery yet needs to be further clarified.

## Conflicts of interest

The authors have no conflicts of interest to report.

## Funding

This manuscript has been written with no funding involved.
